# The kinetics of lactate production and removal during whole-body exercise

**DOI:** 10.1186/1742-4682-9-7

**Published:** 2012-03-13

**Authors:** John F Moxnes, Øyvind Sandbakk

**Affiliations:** 1 Department for Protection Norwegian Defence Research Establishment, P.O. Box 25, 2007 Kjeller, Norway; 2 Department of Human Movement Science, Norwegian University of science and Technology, 7491 Trondheim, Norway

**Keywords:** Aerobic power, Anaerobic power, Blood lactate, Cross-country skiing, Muscle lactate, pH

## Abstract

**Background:**

Based on a literature review, the current study aimed to construct mathematical models of lactate production and removal in both muscles and blood during steady state and at varying intensities during whole-body exercise. In order to experimentally test the models in dynamic situations, a cross-country skier performed laboratory tests while treadmill roller skiing, from where work rate, aerobic power and blood lactate concentration were measured. A two-compartment simulation model for blood lactate production and removal was constructed.

**Results:**

The simulated and experimental data differed less than 0.5 mmol/L both during steady state and varying sub-maximal intensities. However, the simulation model for lactate removal after high exercise intensities seems to require further examination.

**Conclusions:**

Overall, the simulation models of lactate production and removal provide useful insight into the parameters that affect blood lactate response, and specifically how blood lactate concentration during practical training and testing in dynamical situations should be interpreted.

## Background

The metabolic power in humans is based on the production and consumption of adenosine triphosphate (ATP). Despite the approximately 100-fold increase in ATP utilization from rest to maximal-intensity exercise, the energetic demands of the muscles are usually satisfied without depleting the intracellular ATP e.g., [1-3]. In this connection, three sources for ATP synthesis are available; First, ATP can be produced aerobically in the mitochondria by oxidative phosphorylation. Second, ATP can be produced by anaerobic synthesis due to glycolysis or glycogenolysis. Finally, ATP can be produced by phosphocreatine (PCr) break down to Creatine (Cr) (i.e., ADP + PCr gives ATP + Cr in the Creatine Kinease (CK) reaction) e.g., [1-3].

The rate of oxygen (O_2_) consumption can be set to the sum of 1) a constant rate (resting O_2 _consumption), 2) a rate due to unloaded body movements and 3) a rate proportional to the aerobic energy used to perform work. For moderate constant work rates, the aerobic power increases towards a steady state condition. The concept of maximal lactate steady state (MLSS), that is the highest intensity where a steady state lactate can be obtained, has been regarded as important for endurance performance e.g., [4-6]. For exercise intensities above MLSS, associated with sustained acidosis, a slow component delays the attainment of a steady state value and causes O_2 _uptake to increase to values greater than those predicted from aerobic steady state demands. For exercise intensities exceeding the maximal oxygen uptake, the steady state corresponds to the level that would be attained if it was possible to carry out the exercise under pure aerobic conditions [7]. Obviously, this virtual steady state is never reached as the increase in oxygen uptake ends when the maximal oxygen uptake is achieved.

When the rate of ATP production by oxidative sources becomes insufficient, high rates of glycolytic or glycogenolytic ATP production are required. The endpoint of glycolysis is pyruvate, which represents a metabolite that can be reduced to lactate or oxidized to CO_2 _or H_2_O. Thus, by increasing exercise intensities, the working muscles and various tissues produce more lactate and release it into the plasma. At the same time, the skeletal muscles, the heart, the liver and the kidney cortex remove lactate from the circulation, and lactate is suggested to act as an intermediate for the shuttling of carbohydrate from cells and tissue with relatively low oxidative capacity to cells and tissues with high oxidative capacity [8-11]. Thus, it is well established that the blood lactate concentration is the result of the production and the removal of lactate in the blood. During steady state sub-maximal exercise, when lactate production (influx) equals lactate removal (outflux), the lactate concentration in the lactate pool stays constant and the rate of oxygen consumption is the measure of the whole body energy expenditure regardless of the magnitude of lactate production and removal or the absolute blood lactate concentration. At exercise intensities above steady state, a rise in the concentration could be attributed to an increase in the rate of lactate production or result from a decrease in the rate of lactate removal.

Lactate itself does not lead to muscle fatigue at high exercise intensities, and fatigue is most likely a result of decreased muscle pH and the associated reactions e.g., [12,13]. For the skeletal muscles, the pH is normally around 7.1 but can fall to 6.4 during heavy exercise [14], and numerous experiments suggest a negative relationship between decreased pH and the muscle contractile function [15-20]. In the capillary blood pH is normally (in steady state conditions) around 7.45, but can fall to around 7.05 during heavy exercise [21]. Previous researchers have found a close relationship with a time delay between lactate concentrations in muscle and blood [22].

Because muscle groups often work unequally, heterogeneity with regard to lactate concentration is likely in muscles during dynamic exercise. A rigorous estimation would require application of several compartments with production, removal and exchange between compartments. As an approximation, a two-compartment model can be applied, where the muscles and other organs that can remove lactate (such as the heart, the liver and the kidney cortex) are regarded as one compartment and the blood space and other tissues are grouped into a second compartment [23-25]. These studies indicate that the flux of lactate into a compartment depends upon the lactate gradient and its permeability. Furthermore, blood lactate recovery curves from muscular exercise can be described by a bi-exponential time function and a two-compartment model consisting of the previously worked muscle and the remainder lactate space. The time constants of the bi-exponential time function fitted to the arterial blood lactate recovery curves reflect the abilities to exchange lactate between the two-compartments and the ability to remove lactate from the total lactate space including the working muscle department [26]. The two time constants are found to decrease with work rate and duration of the preceding exercise [27,28].

The maximum anaerobic energy that can be utilized is proportional to the sum of Cr and lactate that can be accumulated in the body. PCr is an energy buffer that supports the transient failure of other metabolic pathways to support ATP. The equilibrium constant of the CK reaction is around 20 and the slightest drop in ATP allows the reaction to proceed to ATP. Thus, the ATP concentration stays nearly constant until almost all the PCr is utilized. The PCr levels follow an exponential time course after changes in work rate before approaching a steady state condition at moderate exercise intensities. In such cases, a strong similarity has been reported for the time constants of the O_2 _kinetics and the PCr consumption [29]. However, for exercise intensities above lactate threshold, the anaerobic glycolytic energy supply becomes significant and the association between PCr and O_2 _rate has not yet been systematically reported. During recovery, the level of PCr must be recovered, the pH must be re-established and ADP removed. While the PCr recovery is mainly due to oxidative ATP synthesis, the PCr stores may be rebuilt by anaerobic glycolysis [30-32].

The current study aimed to derive mathematical models for production and removal of lactate in the blood and muscles during dynamic whole-body exercise. As cross-country skiing is a whole body exercise where athletes both train and compete on varying terrain and at constantly varying speeds and work rates [33-35] this locomotion was used for that purpose. Thus, the mathematical models were compared with experimental results from the laboratory during treadmill roller skiing. We hypothesized a mathematical two-compartment model of lactate production and removal to accurately predict blood lactate concentration during steady state and at varying exercise intensities.

## Methods

### Overall design

Initially, the current study derived mathematical simulation models of lactate production and removal in the blood and muscles by utilizing Mathematica 8 (Wolfram Research Inc., Champaign, IL, USA). Thereafter, the simulations were compared with experimental data from an elite skier performing laboratory tests while treadmill roller ski skating both during a steady state and at varying exercise intensities (see details below).

### Steady state aerobic power

Work rate W on the treadmill is $=\underset{\approx 0}{\underset{︸}{mv\dot{v}}}+\mu mgCos\left(\alpha \right)v+mgSin\left(\alpha \right)v$, where v is the treadmill velocity, *μ *the coefficient of friction, m the mass of the skier and *α *≈ *Sin*(*α*) is the treadmill incline in radians. The power due to the change of kinetic energy $mv\dot{v}$ is zero on the treadmill since the velocity is constant. *μmgCos*(*α*)*v *is the power of roller friction, and *mgSin*(*α*)*v *is the power of gravity due to the inclination of the treadmill. We define *Q*_*max *_to be the maximal aerobic power and ${\bar{Q}}_{a}$ to be the steady state aerobic power that has been found while treadmill roller skiing. $\overset{≂}{Q}a$ is regarded as the virtual steady state aerobic power, with ${\bar{Q}}_{a}\overset{mod}{=}Min\left({\bar{\tilde{Q}}}_{a},Q_{max}\right)$, where *Min *is the minimum function. The *Min *function is employed to ensure that the aerobic power does not exceed the maximum aerobic power. Below *Q*_*max *_the virtual steady state power ${\bar{\tilde{Q}}}_{a}={\bar{Q}}_{a}$ is found to be linear with work rate for a given cycle rate and incline, and as a hypothesis we apply this linearity also for metabolic powers above *Q*_*max *_and find

(1) $$\begin{array}{c} \;\;\;\;{\bar{\tilde{Q}}}_{a}\overset{mod}{=}\underset{rest}{\underset{︸}{Q_{b}}}+\underset{unloaded}{\underset{︸}{Q_{ul}\left(f\right)}}+c_{2}\times \underset{loaded}{\underset{︸}{Ā\left(\alpha \right)}}\times W,{\bar{Q}}_{a}\overset{mod}{=}Min\left({\bar{\tilde{Q}}}_{a},Q_{max}\right)\\ Q_{b}=80J/s,Q_{ul}\left(f\right)\approx 111J/s,c_{2}=5.8,Ā\left(\alpha \right)\overset{def}{=}0.92\left(1+1.19Exp\left(-71.8\alpha \right)\right) \end{array}$$

*Q*_*b *_is the metabolic power at rest (set to 80 J/s), *Q*_*un *_the metabolic power of unloaded movements (zero work rate) which is dependent of the cycle rate. We define the cycle rate to be constant in this article. Thus, *Q*_*un *_= 111 J/s here.

Thereafter, we let *Q*_*a *_be the aerobic power, ${\tilde{Q}}_{a}$ the virtual aerobic power and $Q_{a}\overset{mod}{=}Min\left(Q_{max},{\tilde{Q}}_{a}\left(t\right)\right)$. A first order differential equation of aerobic power with the virtual aerobic power as input was used to account mathematically for the delay in aerobic power with a time lag during steady state work rate. Thus,

(2) $$\begin{array}{c} {\dot{\tilde{Q}}}_{a}\left(t\right)\overset{mod}{=}\frac{{\bar{\tilde{Q}}}_{a}-{\tilde{Q}}_{a}\left(t\right)}{\tau_{a}},\left(a\right),Q_{a}\overset{mod}{=}Min\left(Q_{max},{\tilde{Q}}_{a}\left(t\right)\right),\left(b\right),\tau_{a}=\\ 30s,Q_{a}\left(t_{0}\right)=Q_{b} \end{array}$$

The "dot" means time derivative, *τ *is a time parameter quantifying the time before the aerobic power reaches steady state during sub-maximal work rates. We uses *τ *= 30 s according to di Prampero [36].

**Figure 1:**
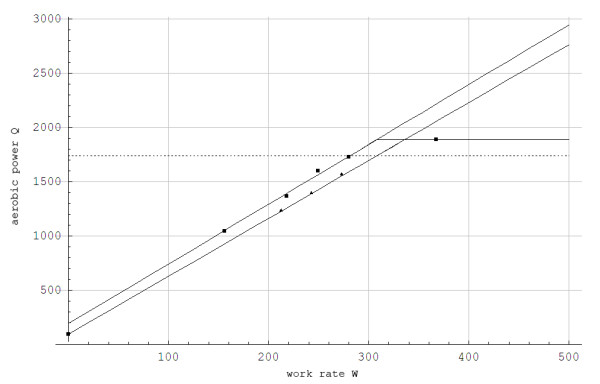
**The aerobic power Q(W, α) as a function of work rate (W) for inclines of α = 0.05 (upper line) and α = 0.12 (lower line) during treadmill roller skiing using the skating technique**. **The curve fittings are based on least square fit to the data. Maximal metabolic rate (Q_max _= 1886 J/s) is represented by a straight horizontal line**. The lactate threshold (Q_LT _= 1755 J/s) is represented by the straight dotted horizontal line. ■: Experimental values for α = 0.05, ▲: Experimental values for α = 0.12.

Figure 1 shows the steady state aerobic power as function of the work rate for the G3 skiing technique.

### Anaerobic power and lactate concentration in blood and muscle

Lactate concentration in the lactate pool (*C*(*t*)), i.e. the mass of lactate per unit volume of this pool, increases only if the rate of lactate appearance (influx) in the lactate pool is larger than the rate of lactate disappearance (outflux). The current study uses a modified version of Brooks [8] and determines the levels of lactate pool concentration by modeling both the influx and outflux streams of lactate and simulate the blood lactate concentration according to Moxnes and Hausken [37]. Here two pyruvate molecules are produced for each glucose or glycogen molecule during glycolysis/glycogenolysis. One molecule of pyruvate gives one molecule of lactate. The increase in glycogenolysis/glycolysis, due to increased exercise intensity, ends when a maximal rate of glycogenolysis/glycolysis is achieved [37]. Therefore, the rate of pyruvate appearance (P) has a least upper bound, which is denoted as *P*_*max*_. By neglecting rates due to changing pyruvate concentration in the plasma, the rate of lactate appearance in mmol/L is *R*_*a *_= *P*. However, as pyruvate can be oxidized in the mitochondria we set the influx of pyruvate into the mitochondria to be *α*_0_*Tanh*(*β*_0_, *P*)/(*β*_0_, *α*_0 _< 1, where *α*_0 _and *β*_0 _are to parameters fitted to the data and Tanh() is a function that accounts for saturation at high lactate concentrations according to Moxnes and Hausken [37]. Thus, the rate of lactate appearance is *R*_*a *_= *P *- *α*_0_*Tanh*(*β*_0_*P*)/*β*_0 _where we expect that *α*_0 _is around 1.

During severe exercise, glycogen re-synthesis by the liver is severely depressed. As a hypothesis, we forecast that the rate of lactate disappearance due to both glycogen re-synthesis and lactate oxidation is *R*_*d *_= *d*_0 _× (*Tanh*(*χ*^*C*^)/*χ*) × *D*(*Q*_*a*_) × (*Q*_*max *_- *Q*_*a*_), where *d*_*0 *_and *χ *are two parameters that are fitted to the data. *D*(*Q*_*a*_(*t*)) is an unknown function that is monotonically increasing with the aerobic power. When aerobic power equals the maximum aerobic power (i.e. *Q*_*a *_= *Q*_*max*_) no lactate disappearance takes place. Altogether, for a one compartment model of lactate we have

(3) $$\begin{array}{c} Ċ\left(t\right)=\\ R_{a}-R_{d}\overset{mod}{=}P-\underset{apperance}{\underset{︸}{\alpha_{0}Tanh\left(\beta_{0}P/\right)}}/\beta_{0}-\\ d_{0}\times \underset{disapperance}{\underset{︸}{\left(Tanh\left(\chi C\right)/\chi \right)\times D\left(Q_{a}\right)\times \left(Q_{max}-Q_{a}\right)}} \end{array}$$

In equation (3) we need a model for the rate of pyruvate appearance (P). To our knowledge, no such model exists in the literature, and we hypothesize that this function is linear with work rate up to *P*_*max *_which is the least upper bound. This simple assumption might constitute a potential weakness in our argument. However, if we have experimental support this assumption also seems like the most reasonable one. Thus, we define a virtual steady state ($\left(\bar{\tilde{P}}\right)$) analogous to the virtual aerobic steady state $\left({\bar{\tilde{Q}}}_{a}\right)$ and hypothesize that

(4) $$\dot{\tilde{P}}\left(t\right)\overset{mod}{=}\frac{\bar{\tilde{P}}-\tilde{P}\left(t\right)}{\tau_{an}},\tau_{an}=10s,P\overset{mod}{=}Min\left(P_{max},\tilde{P}\left(t\right)\right)$$

where *τ*_*an *_is the time constant for full activation of glycolysis/glycogenolysis during muscle contractions, set to 10 s [38]. $\bar{P}\left(t\right)$ is the steady state rate of pyruvate appearance. Note that the model in equation (3) applies only for the chosen type of exercise and a fixed concentration of glycogen in the body.

For aerobic power we assume that $\overset{≂}{Q}a$ is a linear function of work rate and forecast that the steady state rate of pyruvate appearance is approximately proportional to the virtual steady state power leading to

(5) $$\bar{\tilde{P}}\left(t\right)\overset{mod}{=}p_{0}{\bar{\tilde{Q}}}_{a}$$

Due to the rather fast response time in equation (4), an approximation is that $\left(t\right)\approx \bar{\tilde{P}}=p_{0}{\bar{\tilde{Q}}}_{a}\left(t\right)$. For equation (3) this gives that

(6) $$\begin{array}{c} Ċ\left(t\right)\approx p_{0}\left({\bar{\tilde{Q}}}_{a}\left(t\right)-\frac{\alpha_{0}}{\beta_{0}p_{0}}T_{anh}\left(\beta_{0}p_{0}{\bar{\tilde{Q}}}_{a}\left(t\right)\right)\right)-d_{0}\times \left(Tanh\left(\chi C\left(t\right)\right)/\chi \right)\times D\left(Q_{a}\left(t\right)\right)\times \left(Q_{max}-Q_{a}\left(t\right)\right),\\ \;\;\;\;\;\;\;\;\;\;\;\;C\left(t_{0}\right)=0.045kg/m^{3}=0.5mmol/L \end{array}$$

Finally, a model for *D*(*Q*_*a*_) is needed. As a hypothesis we set that

(7) $$D\left(Q_{a}\right)\overset{mod}{=}\propto \left(Q_{a}-\frac{\alpha_{0}}{\beta p_{0}}Tanh\left(\beta_{0}p_{0}Q_{a}\right)\right)$$

Here the constant of proportionality can be scaled by *d*_0_.

A general solution for constant aerobic power is feasible in (5)-(7) when we let $Tanh\left(\chi C\left(t\right)\right)/\chi \approx C\left(t\right)\;\;\left(Q_{a}={\bar{Q}}_{a}=\text{constant}\right)$[37].

Altogether, the steady state lactate concentration for constant aerobic power at intensities below the MLSS from equation (5)-(7) is

(8) $$\begin{array}{l} \bar{C}=ArcTanh\left(\frac{p_{0}\chi }{d_{0}(Q_{max}-{\bar{Q}}_{a})}\right)/\chi =\frac{1}{2}Ln\left(\frac{1+\frac{\chi p_{0}}{d_{0}Q_{max}(1-{\bar{Q}}_{a}/Q_{max})}}{1-\frac{\chi p_{0}}{d_{0}Q_{max}(1-{\bar{Q}}_{a}/Q_{max})}}\right)/\chi \\ \;\;\;\;\;\;\;\;\;\;\underset{\chi \to 0}{Lim\bar{C}}=\frac{p_{0}}{d_{0}(Q_{max}-{\bar{Q}}_{a})} \end{array}$$

The maximum aerobic power that can be used for steady state concentration is given by

(9) $$1-\frac{p_{0}}{d_{0}\left(Q_{max}-{\bar{Q}}_{a}\right)}=0\Rightarrow {\bar{Q}}_{a}={\bar{Q}}_{LT}=Q_{max}-\frac{p_{0}\chi }{d_{0}}\Rightarrow \frac{{\bar{Q}}_{LT}}{Q_{max}}=1-\frac{p_{0}\chi }{d_{0}Q_{max}}$$

where ${\bar{Q}}_{LT}=0.93Q_{max}=\text{MLSS = },\;p_{0}=10^{-5}kg/\left(m^{3}s\right)/\left(J/s\right),d_{0}=7.2410^{-8}/{\left(J/s\right)}^{2}/s,\chi =0.95m^{3}/kg$ and *Q*_*max *_= 1886 J/s.

According to equation (8) all steady state lactate concentrations can be achieved for steady state aerobic powers below ${\bar{Q}}_{LT}$ (MLSS). Furthermore, the steady state lactate concentration $\left(\bar{C}\right)$ approaches infinity when the aerobic power approaches ${\bar{Q}}_{LT}$. However, it has been discovered that not all steady state lactate levels are tolerated over time. This means that blood lactate levels above a certain level of exercise can be terminated before steady state is reached. The anaerobic power due to anaerobic glycogenolysis/glycolysis $\left(Q_{an}^{G}\left(t\right)\right)$ can be calculated from the increase lactate concentration when using the relation from di Prampero and Ferretti [7] as

(10) $$Q_{an}^{G}\left(t\right)=m\times \lambda \times Ċ\left(t\right),\lambda =3\times 20:J/\left(kgmmol/L\right)$$

Blood lactate concentration usually continues to rise a short period of time after exercise. The model in equation (6) does not capture this phenomenon since we only considered one compartment; the lactate pool. In the next step we consider the different compartments involved: working muscles, blood and other tissues such as the liver, kidney and heart. For exercise powers significantly above resting values, we regard a two-compartment model as sufficient: a) the blood compartment and b) muscles and other tissues as one compartment (denoted muscles in the rest of current manuscript). *C*_*b*_(*t*) is the lactate concentration in the blood and *C*_*m*_(*t*) the lactate concentration in the muscles. *V*_*b*_(*t*) and *V*_*m*_(*t*) are the volumes of muscles and blood, respectively. The total lactate pools volume is *V *= *V*_*b*_(*t*) + *V*_*m*_(*t*), set to 0.18 L per kg body mass as an approximation for the skier modelled here [39]. The muscle mass is set to be 10 kg, based on an iDexa scan of the skier, and muscle volume as 10 L. We assume that lactate moves between the muscle and blood compartment with some time dynamics. Thus, we propose the following model for the muscle's (C_m_) and blood's (C_b_) concentrations of lactate

(11) $$\begin{array}{l} {\dot{C}}_{m}(t)=\left(\frac{V}{V_{m}}\right)\left[p_{0}D\left({\bar{\tilde{Q}}}_{a}(t\right)-d_{0}(Tanh(\chi C_{m}(t))/\chi )D(Q_{a}(t))(Q_{max}-Q_{a})\right]-k_{1a}\frac{C_{m}(t)-C_{b}(t)}{V_{m}}\\ \;\;\;\;\;\;\;\;\;\;\;\;\;{\dot{C}}_{b}(t)=k_{1a}\frac{C_{m}(t)-C_{b}(t)}{V_{b}} \end{array}$$

*K*_1*a *_is a parametric function that scales the movement of lactate into and out of the blood. The rate of lactate appearance in the blood is set proportional to the difference in lactate concentration between blood and muscles. To account for different rates of transport into or out of the blood we let *K*_1*a *_be dependent on *C*_*m*_(*t*) - *C*_*b*_(*t*). Estimates of these parameters were found by visual curve fitting. Visual curve fitting gives plausible values for the parameters based on plotting of experimental data that is compared with simulations. The parameters were sought to have biologically trustworthy numerical values and a least square fit of the data was performed to produce best fit estimates. The parameters employed here are

(12) $$\begin{array}{c} p_{0}=10^{-5}kg/\left(m^{3}s\right)/\left(J/s\right),d_{0}=7.2410^{-8}/{\left(J/s\right)}^{2}/s,\chi =0.95m^{3}/kg,\alpha_{0}=0.9\\ \;\;1/\left(\beta_{0}p_{0}\right)=0.6Q_{max},\alpha_{0}=0.9,k_{1a}=0.05/s\times L,V_{b}=4L,V_{m}=10L \end{array}$$

### Experimental tests

The derived mathematical simulations are compared with experimental data from an elite crosscountry skier while roller skiing on a treadmill employing the skating G3 technique. The mass of the skier was m = 77.5 kg and the friction coefficient on the treadmill was *μ *= 0.024 in all tests. Equipment and procedures were similar to the studies by Sandbakk, Holmberg, Leirdal and Ettema [40,41]. All treadmill tests were performed on a 6 × 3 m motor-driven treadmill (Bonte Technology, Zwolle, The Netherlands). Inclination and speed were calibrated using the Qualisys Pro Reflex system and the Qualisys Track Manager software (Qualisys AB, Gothenburg, Sweden). The treadmill belt consisted of a non-slip rubber surface that allowed the skier to use his own poles (pole length: 90% of body height) with special carbide tips. The skier used a pair of Swenor skating roller skis with standard wheels (Swenor Roller skis, Troesken, Norway) and the Rottefella binding system (Rottefella AS, Klokkartstua, Norway), and the roller skis were pre-warmed before each test through 20-min of roller skiing on the treadmill. The roller skis were tested for rolling friction force (F_f_) before the test period, and the friction coefficient (μ) was determined by dividing F_f _by the normal force (N) (μ = F_f _· N^-1^). This was performed in a towing test on three subjects (70, 80 and 90 kg) rolling passively at 3.9, 4.4 and 5.0 m/s for 5 min on a flat treadmill (0%) whilst connected to a strain gauge force transducer (S-type 9363, Revere Transducers Europe, Breda, The Netherlands). The measured μ was independent of speed and body mass, and the mean μ-value (0.0237) was incorporated in the work rate calculations. Gas exchange values were measured by open-circuit indirect calorimetry using an Oxycon Pro apparatus (Jaeger GmbH, Hoechberg, Germany). Before each measurement, the VO_2 _and VCO_2 _gas analyzers were calibrated using high-precision gases (16.00 ± 0.04% O_2 _and 5.00 ± 0.1% CO_2_, Riessner-Gase GmbH & co, Lichtenfels, Germany), the inspiratory flow meter was calibrated with a 3 L volume syringe (Hans Rudolph Inc., Kansas City, MO). Heart rate (HR) was measured with a heart rate monitor (Polar S610, Polar Electro OY, Kempele, Finland), using a 5-s interval for data storage. Blood lactate concentration (BLa) was measured on 5 μL samples taken from the fingertip by a Lactate Pro LT-1710 *t *(ArkRay Inc, Kyoto, Japan).

In a first test, the skier performed 5-min constant work rates at 0.05 and 0.12 inclines in radians when treadmill roller skiing in the skating G3 technique. Gas exchange values were determined by the average of the last minute during each stage. The lactate threshold was defined at the metabolic power when blood lactate began to accumulate (OBLA) (defined as a concentration of 4 mmol/L, as calculated by a linearly interpolated point out of the three measurement points of blood lactate concentration at the incline of 0.05. Maximal metabolic power was tested at an incline of 0.05 in the G3 technique with a starting speed of 4.4 m/s. The speed was increased by 0.3 m/s every minute until exhaustion. VO_2 _was measured continuously, and the average of the three highest 10-s consecutive measurements determined VO_2_max and used to calculate the maximal metabolic power. The test was considered to be a maximal effort if the following three criteria were met: 1) a plateau in VO_2 _was obtained with increasing exercise intensity, 2) respiratory exchange ratio above 1.10, and 3) blood lactate concentration exceeding 8 mmol/L.

**Figure 2:**
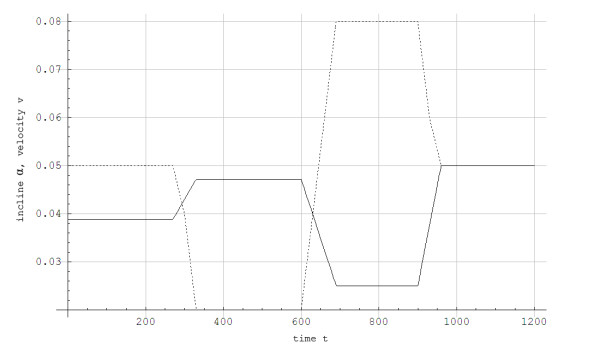
**Velocity and incline as a function of time while treadmill roller skiing using the skating G3 technique**.___: The skier's velocity (v) in 10^2 ^m/s as a function of time (t) in seconds....:The treadmill's incline (α) in radians as a function of time (t) in seconds.

Thereafter, the velocity and the angle of incline on the treadmill were varied according to Figure 2 with gas exchange measured continuously. Immediately after finishing the protocol in Figure 2, the skier had a recovery period while skiing at 0.05 incline at 2.2 m/s, inducing a work rate of 125 J/s and an aerobic power of approximately 900 J/s.

## Results

**Figure 3:**
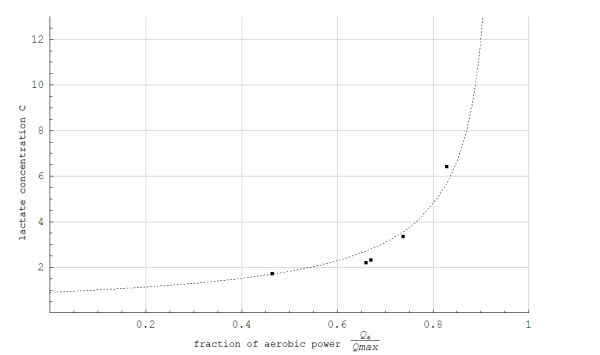
**Steady state concentrations of blood lactate (C) in mmol/L as a function of the fraction of maximum aerobic power while treadmill roller skiing using the skating G3 technique at a 0.05 incline**. ■: Experimental data ------: $\text{C}=\bar{\text{C}}=\frac{1}{2}\text{Ln}\left(\frac{1+\frac{\chi {\text{p}}_{0}}{{\text{d}}_{0}{\text{Q}}_{\text{max}}\left(1-{\text{Q}}_{\text{a}}/{\text{Q}}_{\text{max}}\right)}}{1-\frac{\chi {\text{p}}_{0}}{{\text{d}}_{0}{\text{Q}}_{\text{max}}\left(1-{\bar{\text{Q}}}_{\text{a}}/{\text{Q}}_{\text{max}}\right)}}\right)/\chi$.

**Figure 4:**
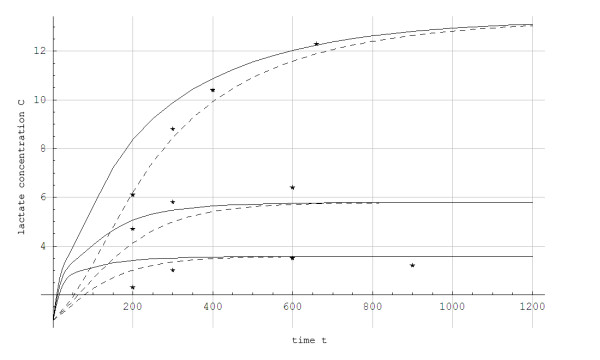
**The concentration of blood and muscle lactate concentration (C) in mmol/L for a skier while treadmill roller skiing in the skating G3 technique at a 0.05 incline at velocities of 3.89 m/s, 5 m/s and 7.44 m/s, respectively**. * = experimental data, ___: Muscle compartment simulation, --------: Blood compartment simulation.

**Figure 5:**
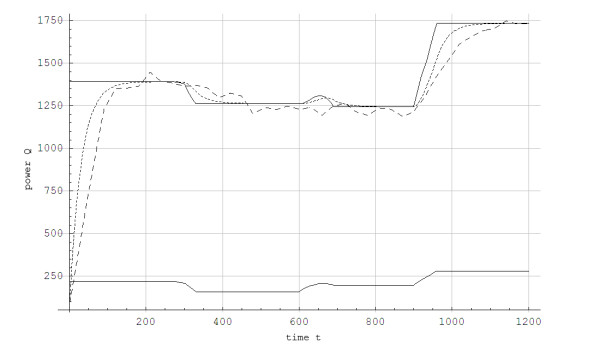
**Calculations of work rate (W) and metabolic powers (Q) as functions of time (t) in J/s for a skier while treadmill roller skiing in the skating G3 technique at the velocities and inclines shown in Figure 2**. _____: Upper ${\bar{Q}}_{a}\left(t\right)$,____: Lower: Work rate (W), - - - - -: Experimental data, .........:Q_a_(t).

**Figure 6:**
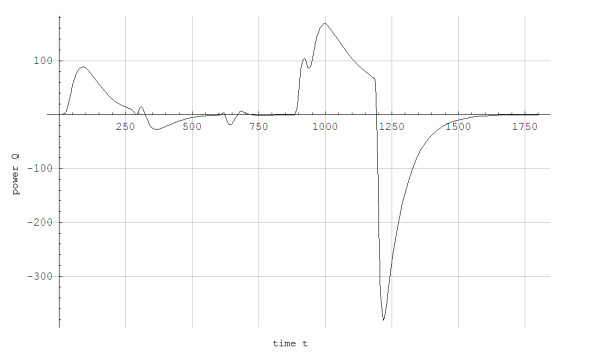
Simulated anaerobic power (Q) in J/s as a function of time (t) in seconds for a skier while treadmill roller skiing in the skating G3 technique at the velocities and inclines shown in Figure 2 and a subsequent recovery period at 0.05 incline at 2.2 m/s.

**Figure 7:**
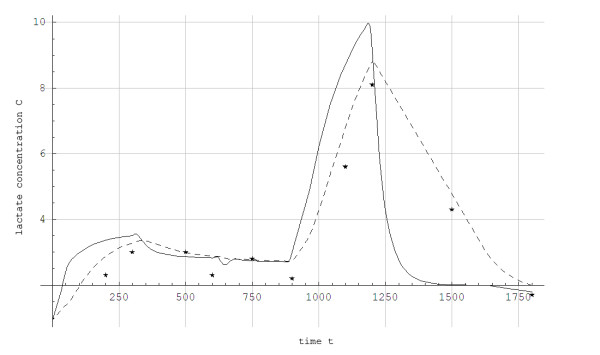
**Simulated blood and muscle lactate concentrations (C) in mmol/L as a function of time (t) in seconds for a skier while treadmill roller skiing in the skating G3 technique at the velocities and inclines shown in Figure 2 and a subsequent recovery period at 0.05 incline at 2.2 m/s**. *: Experimental data, ___: Muscle compartment simulation, -------: Blood compartment simulation.

The simulated data for steady state concentration of blood lactate during 5-min constant work rates show good agreement with the experimental results, with less than 0.5 mmol/L disagreement (Figures 3 and 4). For the protocol showed in Figure 2, the simulated and experimental data for work rate and metabolic power as functions of time show general good agreement (Figure 5). However, we see that *Q*_*a*_(*t*) is delayed compared to the quasi steady state value ${\bar{Q}}_{a}\left(t\right)$. Additionally, *Q*_*a*_(*t*) is delayed compared to the experimental data because the measuring apparatus of VO_2 _has an inherent time lag (around 15 s) that is not modeled. Based on the same test (i.e., Figure 2), anaerobic power is simulated in Figure 6. Thereafter simulated and experimental lactate levels as functions of time were compared in Figure 7. There was a good agreement between the simulated and experimental results at these varying sub-maximal intensities, showing less than 0.5 mmol/L disagreements. During the recovery period after performing the protocol in Figure 2, the initial simulation model of the concentrations of blood and muscle lactate did not fit well with experimental results. The experimental data showed a much slower reduction in blood lactate concentration compared to the simulated model. Thus, in a second trial we assumed a maximum rate of flux of lactate from blood to muscles. We achieved this mathematically when *C*_*m*_(*t*) - *C*_*b*_(*t*) → *Max*[-0.1, *C*_*m*_(*t*) - *C*_*b*_(*t*)] which accounts for a restriction on the lactate flux from blood to muscles. This means that the flux of lactate from the blood to the muscles has a least upper bound. The value of -0.1 was the value that fitted the experimental data best by visual inspection. Using this assumption, the simulated and experimental data were in much better agreement (see Figure 7).

## Conclusions

The current study constructed mathematical models of lactate production and removal and compared these with experimental results from treadmill roller skiing. The main findings were as follows: 1) a mathematical two-compartment model of lactate production and removal could accurately predict blood lactate concentration during steady state and at varying exercise intensities and; 2) the understanding of lactate removal after high-intensity exercise during whole-body exercise requires further examination.

In the current study, the simulated lactate production and removal fitted the experimental data well during steady state and at varying sub-maximal exercise intensities, and indicate an uncertainty of less than ± 0.5 mmol/L using these mathematical models. To the best of our knowledge, the current study is the first to compare a simulation model of blood lactate concentration during dynamic whole-body exercise against experimental data. Overall, we propose that the current simulation models provide useful insight into how blood lactate concentration during practical training and testing in dynamical situations should be interpreted.

The initial simulation model of lactate removal after high exercise intensities deviated from the experimental findings. This can be related to a longer time delay between lactate concentrations in muscle and blood than simulated by the model [22] or a slower removal of blood lactate than the bi-exponential time function employed here [26-28]. In any case, when assuming a maximum rate of flux of lactate from blood to muscles did the simulation model fit experimental data satisfactorily. A rationale for this asymmetry of the time scale for influx and outflux of lactate from the blood may be related to the transport of lactate across membranes accomplished by monocarboxylate transport proteins (MTC). MCT are used for active transport of lactate and is driven by concentration gradients [9,13]. However, the physiological role of the two important isoforms MCT1 and MCT4 differ. MCT1 is mainly located in slow twitch muscle fibers and is regarded responsible for the influx of lactate to the high oxidative muscles, whereas MCT4 is mainly located in fast muscle fibers and is responsible for outflux of lactate from cells. The current data indicate that the amount of MTC4 is low in blood cells, which leads to a smaller outflux of lactate from the blood during recovery than the influx of lactate to the blood during high intensity exercise. Nevertheless, the understanding of lactate removal after high-intensity exercise during whole-body exercise requires further examination.

Modeling in human biology is always a challenge since one is confronted with conceiving a simple, yet realistic representation of complex phenomena occurring at different levels (cells, organs, tissue). The parameters used in the current study will, in principle, be dependent on the exercise mode employed; fitness level of the individual tested and is only applicable for fixed concentrations of glycogen e.g., [42-44]. Thus, to construct valid simulation models of lactate concentration during dynamic exercise these need to be developed for each individual.

## Abbreviations

W, Work rate in J/s; v, Velocity of the treadmill in m/s; α, Angle of inclination in radians; m = 77.5 kg, Mass of the skier; μ = 0.024, Coefficient of roller friction on the treadmill; g = 9.82 m/s2, Acceleration of gravity; *Q*_*max *_= 1886, Maximal aerobic power in J/s; ${\bar{Q}}_{LT}=0.93\text{Qmax}$:, Aerobic power at the lactate threshold relative to the maximal aerobic power; Qa, Aerobic power in J/s; ${\bar{Q}}_{a}$, Steady state aerobic power in J/s; ${\tilde{Q}}_{a}$, Virtual aerobic power in J/s; $\overset{≂}{Q}a$, Virtual steady state aerobic power in J/s; Qb = 80 J/s, Resting aerobic power; *Q*_*a *_(*t*_0_) = *Q*_*b*_, Initial aerobic power; Qul = 111 J/s, Aerobic power of unloaded movement of arms and legs; *f*, Cycle frequency of the skier in the G3 technique in 1/s; Ā(α)=0.92(1+1.19Exp(-71.8α)), Function that describes the influence of incline on ${\bar{\tilde{\text{Q}}}}_{\text{a}}$; *c*_2 _= 5.8 J/s, Parameter that describes the influence of work rate on ${\bar{\tilde{\text{Q}}}}_{\text{a}}$; *τ *= 30 s, Time parameter in seconds quantifying the time before the aerobic power reaches steady state during sub-maximal work rates; *C*, Lactate concentration in the lactate pool in *kg*/*ml *or in *mmol*/*L*; $\left(\bar{C}\right)$, Steady state lactate concentration in the lactate pool in *kg*/*ml *or in *mmol*/*L*; *C*(*t*_0_) = 0.045*kg*/*m*^3 ^= 0.5*mmol*/*L*, Initial concentration of lactate; *R*_*a*_, Rate of lactate appearance in kg/(sm^3^) or in mmol/(sL); *R*_*d*_, Rate of lactate disappearance in kg/(sm^3^) or in mmol/(sL); *P*_*max*_, Maximum rate of pyruvate appearance in kg/(sm^3^) or in mmol/(sL); *P*, Rate of pyruvate appearance in kg/(sm^3^) or in mMol/(sL); $\bar{P}$, Steady state rate of pyruvate appearance in kg/(sm^3^) or in mmol/(sL); $\tilde{P}$, Virtual rate of pyruvate appearance in kg/(sm^3^) or in mmol/(sL); $\left(\bar{\tilde{P}}\right)$, Virtual steady state pyruvate appearance in kg/(sm^3^) or in mmol/(sL); *τ*_*an *_= 10 s, Time parameter quantifying the time before the pyruvate appearance reaches steady state during sub-maximal work rates; *α*_0 _= 0.9, Parameter that describe the rate of pyruvate disappearance due to oxidation in the mitochondria; *β*_0 _= 1/(0.6 × *p*_0 _× *Q*_*max*_), Parameter that describes the rate of pyruvate disappearance due to oxidation in the mitochondria; *p*_0 _= 10^-5^*kg*/(*m*^3^*s*)/(*J*/*s*), Parameter that describes the rate of lactate appearance as a function of aerobic power; *D*(*Q*_*a*_(*t*)), Function that describes the rate of lactate disappearance as a function of aerobic power; *d*_0 _= 7.2410^-8^/(*J*/*s*)^2^/*s*, Parameter that describes the rate of lactate disappearance as a function of aerobic power; *χ *= 0.95*m*^3^/*kg*, Parameter that describes the saturation of disappearance of lactate; $Q_{an}^{G}$, Power caused by the use of ATP produced anaerobically from glycolysis or glycogenolysis; *λ *= 3 × 20:*J*/(*kgmmol*/*L*), Parameter that scales the rate of change in lactate concentration and anaerobic power; *V*_*m *_= 10*L*, Lactate volume of the muscles; *V*_*b *_= 4*L*, Lactate volume of the blood; *V *= *V*_*b *_+ *V*_*m*_, Total lactate volume; *K*_1*a *_= 0.05/s × *L*, Parameter that scales the movement of lactate into and out of the blood

## Competing interests

The authors declare that they have no competing interests.

## Authors' contributions

JM performed the mathematical simulations, whereas ØS performed all laboratory testing. Both authors contributed similarly with important intellectual content and in drafting, revising and finishing the manuscript. All authors read and approved the final manuscript.

## Funding

No sources of funding.
